# GIS-Based Multi-Criteria Analysis for Arabica Coffee Expansion in Rwanda

**DOI:** 10.1371/journal.pone.0107449

**Published:** 2014-10-09

**Authors:** Innocent Nzeyimana, Alfred E. Hartemink, Violette Geissen

**Affiliations:** 1 Soil Physics and Land Management Group, Wageningen University, Wageningen, The Netherlands; 2 Department of Soil Science, FD Hole Soils Lab, University of Wisconsin, Madison, Madison, Wisconsin, United States of America; Tennessee State University, United States of America

## Abstract

The Government of Rwanda is implementing policies to increase the area of Arabica coffee production. Information on the suitable areas for sustainably growing Arabica coffee is still scarce. This study aimed to analyze suitable areas for Arabica coffee production. We analyzed the spatial distribution of actual and potential production zones for Arabica coffee, their productivity levels and predicted potential yields. We used a geographic information system (GIS) for a weighted overlay analysis to assess the major production zones of Arabica coffee and their qualitative productivity indices. Actual coffee yields were measured in the field and were used to assess potential productivity zones and yields using ordinary kriging with ArcGIS software. The production of coffee covers about 32 000 ha, or 2.3% of all cultivated land in the country. The major zones of production are the Kivu Lake Borders, Central Plateau, Eastern Plateau, and Mayaga agro-ecological zones, where coffee is mainly cultivated on moderate slopes. In the highlands, coffee is grown on steep slopes that can exceed 55%. About 21% percent of the country has a moderate yield potential, ranging between 1.0 and 1.6 t coffee ha^−1^, and 70% has a low yield potential (<1.0 t coffee ha^−1^). Only 9% of the country has a high yield potential of 1.6–2.4 t coffee ha^−1^. Those areas are found near Lake Kivu where the dominant soil Orders are Inceptisols and Ultisols. Moderate yield potential is found in the Birunga (volcano), Congo-Nile watershed Divide, Impala and Imbo zones. Low-yield regions (<1 t ha^−1^) occur in the eastern semi-dry lowlands, Central Plateau, Eastern Plateau, Buberuka Highlands, and Mayaga zones. The weighted overlay analysis and ordinary kriging indicated a large spatial variability of potential productivity indices. Increasing the area and productivity of coffee in Rwanda thus has considerable potential.

## Introduction

Coffee is one of the most important tradable commodities in the world and a major foreign-exchange earner in many developing countries [1]. Arabica coffee accounts for two-thirds of the global coffee market [2]. Coffee is a top export commodity and an important source of revenue in Eastern and Central African countries [3]. In some of these countries, such as Burundi, Uganda, Tanzania, Kenya, and the Democratic Republic of Congo, coffee is occasionally grown in association with agroforestry tree species for nitrogen fixation [4].

Rwanda produces mainly Arabica coffee, largely cultivated by smallholder farmers as mono-crop on plots of less than a hectare scattered on hilly slopes. In South and Central America, coffee is mostly grown on large monoculture plantations or under shade [5]. In Rwanda, coffee is predominantly grown along the shores of Lake Kivu in the west, on the plateau in the central part of Rwanda, and in the Mayaga region in the east [6]. Rwanda has ten agro-ecological zones: Imbo, Impara, Kivu Lake Borders, Birunga (volcano), Congo-Nile Watershed Divide, Buberuka Highlands, Central Plateau, Mayaga-Bugesera, Eastern Plateau, and Eastern Savanna. Details of the characteristics of the Rwandan agro-ecological zones can be found in [7].

The total area of arable and permanently cropped land in Rwanda is about 1.45 million ha [8], of which about 30 000 ha was under coffee production in 2005 and it increased to 41 762 ha in 2012 [3]. The total area under coffee production in the tropics is about 10.6 million ha [9]. The expansion of land for the production of coffee depends on three main factors: environmental conditions (e.g. topography, soil type, climate, and elevation), practices of agricultural land management, and genetic resources (i.e. coffee varieties) [10]. The growing conditions for Arabica coffee in Rwanda are characterized by an altitude of 1400–1900 m a.s.l., an annual rainfall of 1500–1600 mm, temperatures of 18–22°C, and an average amount of sunlight of 2200–2400 hours per year. Arabica coffee also requires fine-textured soils of at least one-meter with total porosities of 50–60%, a pH of 4.5–6.0, moderate to high sums of basic cations, and 2–5% organic matter [7].

In Rwanda, as in other developing countries, coffee farming is reserved for steep slopes and soils with low fertility [6]. Most of these lands have been degraded by soil erosion and are under pressure from intensive cropping by smallholder farmers. Fertile soils are usually reserved for growing staple food crops, which restricts coffee growing to soils of low fertility. In Rwanda, coffee yields above 2.8 t ha^−1^ are rare [6]. The Government of Rwanda has developed a set of policies for improving farmer livelihoods through an increase of sustainable coffee cultivation. A study of agricultural development in Africa has shown that successes are often linked to a cash-crop component and that food crops will profit as a consequence of improved cash income [11]. The identification of potential production zones for expanding coffee production and the prediction of coffee yields are needed to effectively implement these policies.

The evaluation of land is an essential procedure to assess opportunities, potentials, and limitations that a given parcel land can offer for agricultural purposes [12]. Various approaches of land evaluation with specific methodology have been developed to study land-use suitability [12], [13]. Geographic information systems (GISs) have been used for mapping and analyzing land-use suitability [14]. Various GIS-based models have been developed by various researchers for land-use planning and suitability analysis [14]–[21]. The GIS-based models use geo-spatial and geo-statistical tools to assess the land units and to present the results as suitability maps. The models use multi-criteria evaluative approaches and methods by weights, values, or intensities of preference [14], [22]. Weighted overlay analysis is one such approach of GIS modeling using spatial multi-criteria evaluation [23], [24]. The objectives of this study were: (1) to analyze the spatial distribution of potential production zones for Arabica coffee production and the current productivity levels in the various zones and (2) to predict potential coffee yields and identify potential productivity zones. To achieve the objectives, we developed a model of land evaluation for expanding the production of Arabica coffee in Rwanda based on standard methodologies for land evaluation and geo-spatial analysis.

## Material and Methods

### Data acquisition

Digitized and tabulated data were assembled for the entire country, including 43 digital soil maps (scale 1∶50 000), a digital elevation model (Shuttle Radar Topography Mission (SRTM) at 90×90 m resolution), the coffee production database for 2005, general and administrative maps (2006 versions, scale 1∶50 000), and a digital agro-climatic database containing temperatures, rainfall, altitudes, and agro-climatic zones. The database contained the amount of coffee produced and the number of trees in each administrative sector. Each sector was divided into cells, which are the lowest administrative units within the Republic of Rwanda.

### Data processing: analysis of potential zones for coffee production

The methodology consisted of collecting (on a national scale) data such as soil type and slope gradient and analyzing the spatial distribution of coffee using the spatial-analysis toolset of the ArcGIS software [25]. The methodology aimed at identifying the major production zones based on soil and slope types. The combination of data for coffee distribution and soil was used for identifying the dominant soil types on which coffee is mainly produced and then for estimating the area coverage. The combination of data for coffee distribution and slope was for identifying the dominant slope types on which coffee is mainly produced and then for estimating the area coverage. The spatial distribution of coffee was identified, potential coffee production zones were characterized, and the sizes of the areas of coffee per slope and soil type in the various agro-ecological zones were estimated.

### Multi-criteria analysis to estimate a qualitative Arabica coffee productivity index

**Figure 1:**
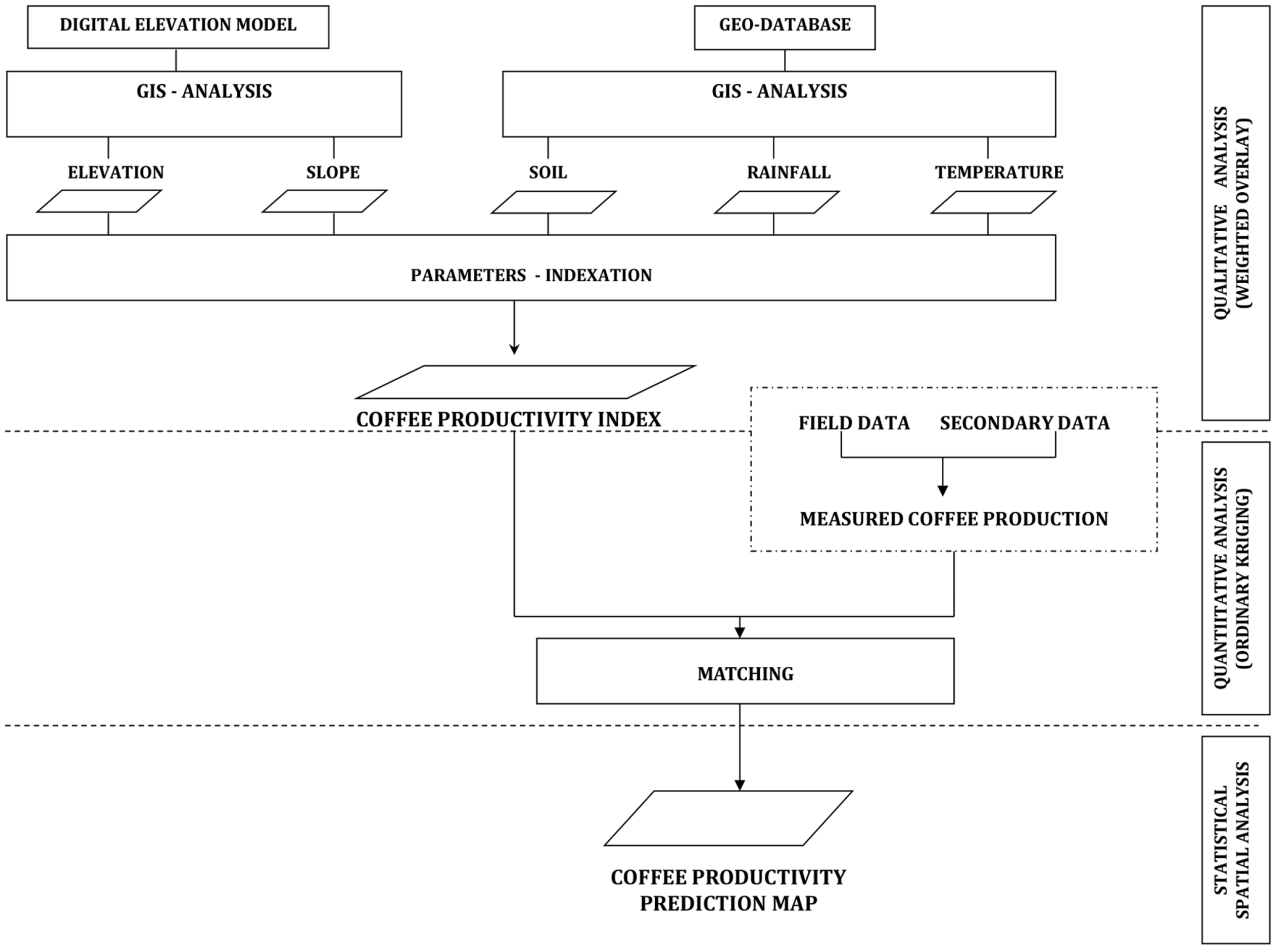
Flow chart of the methodology used to derive coffee productivity indices and predicted Arabica coffee yields.

The assessment of qualitative productivity indices for coffee essentially required the development of a GIS-based database for the optimal use of land resources for coffee. A geo-spatial database of data for elevation, slope, soil parameters, and rainfall and temperature extracted from the digital agro-climatic database, was generated in a GIS multi-criteria model (Figure 1). The landscape characteristics, climatic conditions, and soil parameters of a specific site are the most important determinants of land suitability [17]. The upper part of the flow chart in Figure 1 was thus used to analyze the qualitative productivity indices to indicate the level of productivity in the various agro-ecological zones. The multi-criteria model combined the different layers of data (i.e. elevation, slope, soil parameters, rainfall, and temperature) to identify the major production zones and their current productivity index (CPI). Data for photo synthetically active radiation were not available and so were not included in the model. The multi-criteria analysis used each input raster as a decision variable for sequential GIS interactions between layers. Data were processed using the spatial-analysis tools of ArcGIS [24], [25]. The geo-spatial analysis then allowed the combination of the input rasters using weighted overlay analysis in the Model Builder component of ArcGIS to generate output rasters. Each cell value in each input raster was assigned a new, reclassified score value on an evaluation scale of 1 to 5, where 1 represents the lowest suitability and 5 the highest (i.e. scoring of the Arabica coffee requirements over others based on their importance as guided by [13]). Each of the new reclassified score was then weighted by assigning a percentage influence value (i.e. 100, 75, 50, 25, or 0%) (Table 1). This is achieved by multiplying the cell values (i.e. the new reclassified scores) by their percentage influence, and the results are added together to create the output raster. The new output-raster indices were then used as qualitative productivity indices. The weighted *Z* matrix can have the following form when *m* input factors and *n* criteria are considered:
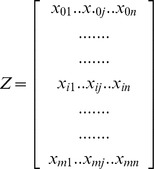
where *Z* is the combined-factor weighted matrix, *m* is the number of input factors, *n* is the number of criteria describing each input factor, *x_ij_* is the score representing the level of importance of input factor *i* based on criterion set *j* of the Arabica coffee requirements. The score *x_ij_* is assigned a percentage influence value according to the importance of the environmental coffee productivity factor within a single input raster as illustrated in Table 1 [24].

**Table 1:** Weighted environmental criteria for evaluating the qualitative Arabica coffee productivity classes and scores (1 to 5 for worst to best)^a^.

Environmental coffee productivity criteria							
Elevation (m)	Rainfall (mm)	Temp. (°C)	Soil type^b^	Slope (%)	Qualitative productivity class	Influence value^c^ (%)	Score^d^
1600–1800	1400–1600	18–20	MOLL, AND	0–4	Very high	100	5
1400–1600 1800–2000	1200–1400 1600–1800	16–18 20–22	ALF	4–12	High	75	4
1200–1400	1000–1200 1800–2000	15–16 22–24	INCEPT, ULT	12–25	Moderate	50	3
1000–1200>2000	800–1000>2000	14–15 24–26	OX, ENT	25–50	Low	25	2
<1000	<800	<14>26	HIST, VERT	>50	Very low	0	1

a The table is a combination matrix that shows the level of productivity if we consider environmental productivity criteria of Arabica coffee production, as guided by [13].

b AND, Andisols; ALF, Alfisols; ENT, Entisols; INCEPT, Inceptisols; HIST, Histosols; MOLL, Mollisols; OX, Oxisols; ULT, Ultisols; VERT, Vertisols.

c The influence value represents the influence of the raster value compared to the other criteria as a percentage (i.e. 100, 75, 50, 25, or 0%).

d Each cell value in each input raster was assigned a new, reclassified score value on an evaluation scale of 1 to 5 (where 5 represented the best score and 1 the worst score 1) The scoring of the environmental productivity criteria of coffee was based on their importance as guided by [13].

The combination of the output rasters is for understanding the influence of the combined environmental factors and/or each factor separately on coffee productivity. The high-productivity class has no significant limitations for sustainable coffee production. The moderate class is characterized by altitudes of 1200–1400 m, annual rainfall below 900 mm or above 2000 mm, and temperatures varying between 18 and 21°C. The low class is characterized by altitudes below 1200 or above 2000 m, annual rainfall below 800 or above 2000 mm, and temperatures below 10 or above 30°C [7], [13].

### Multi-criteria analysis to estimate the quantitative Arabica coffee productivity index

The qualitative productivity indices (low, moderate, and high) were then quantified with actual yields to generate quantitative Arabica coffee productivity indices using ordinary kriging. The qualitative indices were extrapolated to 121 sampled sites of actual Arabica coffee yields measured at various sites countrywide.

### Actual Arabica coffee yield

Actual yields were collected at 121 farms countrywide. Smallholder coffee fields, particularly those near a coffee-washing station, were selected and monitored for yield. The coffee fields are private farms owned by smallholder farmers, technically supported by the National Agricultural Export Development Board (NAEB). In collaboration with the NAEB, the identified farmers participated voluntary in the selection of sample fields. No specific permissions were required for the field activities. In addition, the field studies did not involve any endangered or protected species.

The yields were measured by sampling three branches of coffee trees (low, middle, and high branches). Experimental plots for data collection were approximately 10×10 m and contained 25 coffee trees (i.e. 2500 trees ha^−1^), each 2 m apart. The coffee trees were predominantly 20–25 years of age and were cropped as monocultures.

All sample sites were independently selected with equal probability. Five randomly selected trees in each plot were sampled by collecting a composite sample of 500 g of good berries from the three branches weekly from April to September 2009. The coffee berries were cleaned, oven-dried at 60°C for 48 h, adjusted to 12% moisture content, and weighed. Grain yield was determined on each randomly selected tree, and a spatial mean plot yield was calculated as:
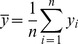
(1)where 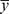 (t ha^−1^) is the average yield for 2009, y_i_ (t ha^−1^) is the yield at sample site i, and n is the number of sample sites.

### Predicted Arabica coffee yield

Ordinary kriging analysis was conducted to predict potential yields and to identify potential productivity zones for Arabica coffee [24], [25], based on actual yields measured at the study site. The ordinary kriging is one of the mostly used geo-statistical methods, quite efficient and accurate for spatial prediction and interpolation [26]. The prediction of yield was based on the qualitative productivity indices validated over the actual yields. A variogram was estimated using Matheron's estimator [27], [28]:
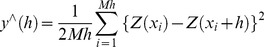
(2)where Z (x_i_) is the actual yield measured at the study site (x_i_), h is the lag, i.e. both distance and direction between the sample sites, M_h_ is the pair of sample sites separated by lag h, and γ∧(h) is the semi-variance at lag h.

To assess the spatial correlation of the yields, prediction accuracy was calculated by comparing expected yields, Z∧(CYI_j_), with actual yields measured at the validation sites, *(n)* - Z*(CYI_j_), and to assess a systematic error, calculated as the mean prediction error (MPE) [29]:
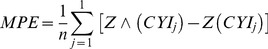
(3)where CYI is the coffee yield index, Z∧(CYI_j_) is the expected yield index generated from the qualitative analysis, and Z*(CYI_j_) is the actual yield measured at the validation sites (n). The validation set accounted for 121 sample sites. The accuracy of prediction was calculated as a root mean square error (RMSE) of prediction [29]:
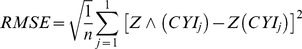
(4)


The RMSE is a measure of fitness of the prediction curve; the smaller the RMSE, the better the prediction. Ordinary kriging uses and compares different fitting models that perform the analysis, reduce uncertainty, and produce the best prediction map. The RMSE is thus standardized by considering the total variance of the observed values and is then termed the root mean square standardized error (RMSSE) or the mean standardized error (MSE). The RMSSE and the MSE were estimated from the variances between the observed values, i.e. the actual yields measured at the study site [24], [29]:
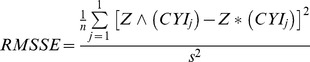
(5)where s^2^ is the total variance of the CYI at the sample site.

A satisfactory accuracy of prediction has an MSE close to zero and an RMSSE close to unity [24], [29]. If the RMSSE exceeds unity, the model underestimates the variability at the validation sites, and thus the prediction is unsatisfactory [24], [29], [30].

The normality of the measured yield data was determined with Kolmogorov-Smirnov test. All data were normally transformed to meet the assumption of normality by comparing different types of model fitting (exponential and Gaussian) for the analysis, and only the model with the smallest Akaike information criterion (AIC) was adopted. The AIC is a measure of how well a model fits the empirical data; the smaller the AIC, the better the fit [24]. In addition, cross-validation, comparing the predicted values with the measured values, checked the quality of the predicted values [31].

## Results

### Spatial distribution of Arabica coffee and biophysical characterization

The estimated area of Arabica coffee production in Rwanda was about 32 000 ha in 2005, compared to 30 000 ha reported by [3]. This area represents about 2.3% of the total area under agriculture. The Central Plateau had the largest area of coffee production, covering about 32% (10 261 ha) of the total area under coffee cultivation. The Central Plateau was characterized by coffee yields of 0.3–2.8 t ha^−1^ (Table 2). This zone has a wide range of soil types and landscapes. The soils where coffee is cultivated included Alfisols, Inceptisols, and Ultisols, representing 4% (1306 ha), 10% (3286 ha) and 12% (3722 ha), respectively, of the total area under coffee (Tables 3 and 4). Cultivated areas in the Central Plateau and the Granitic Ridge agro-ecological zones are also characterized by moderate (<25%) and steep (25–55%) slopes that cover 23% (7212 ha) and 9% (2944 ha) of the total cultivated area, respectively (Table 5). Coffee productivity is mainly limited by infertile soils derived from schistose and granitic materials. Slopes above 25% affect the productivity of the region due to soil erosion.

**Table 2:** Distribution of Arabica coffee areas (ha) and yields (t ha^−1^) calculated from the coffee database for 2005 for the ten agro-ecological zones of Rwanda.

AEZ No.	Agro-Ecological Zone (AEZ)	Total area (ha)	Area with scattered coffee trees^a^ (ha)	Normalized area^b^ (ha)	Range of estimated dry yield^c^ (t ha^−1^)	Mean dry coffee yield ± SD^d^ (t ha^−1^)
1	Imbo (*Lake Kivu region*)	15 832	15 678	804	0.5–2.6	1.2±0.68
2	Impara (*Lake Kivu region*)	64 954	58 532	3376	0.5–2.6	1.2±0.69
3	Kivu Lake Borders (*Lake Kivu region*)	73 593	70 422	2947	0.3–3.5	1.6±0.79
4	Birunga/Volcano	90 887	1952	65	0.5–2.1	1.0±0.74
5	Congo-Nile Watershed Divide	391 930	136 946	4024	0.3–3.5	1.2±0.91
6	Buberuka Highlands	177 154	81 622	1130	0.3–2.8	0.8±0.53
7	Central Plateau & Granitic Ridge	529 772	461 743	10 155	0.3–2.8	0.8±0.47
8	Mayaga-Bugesera *(eastern region*)	223 573	166 085	3328	0.5–1.8	1.0±0.41
9	Eastern Plateau *(eastern region*)	381 367	350 233	5398	0.3–2.2	0.8±0.65
10	Eastern Savana *(eastern region*)	479 761	134 125	692	0.5–2.2	1.1±0.49
	Total	2 564 255	1 162 338	31 921		1.0±0.65

a This area is calculated as the area for each sector in the AEZ; only the sector area in the AEZ is extracted by spatial-analysis tools. The 2005 Rwanda coffee database displays only the number of coffee trees and coffee production per sector. Each sector is an administrative entity divided into cells, which are the lowest administrative units within the Republic of Rwanda.

b This area is extracted from the area with scattered coffee trees in each AEZ and is calculated using the standard tree density of 2500 trees ha^−1^, i.e. b  =  a/2500.

c This yield is calculated by averaging the yields for each part of the sector in the AEZ (i.e. sector yield is calculated as the production of each sector divided by the number of trees using the standard spacing of 2× 2 m, or 2500 trees ha^−1^).

d This yield is calculated using SPSS descriptive statistics; the normality of the data was determined using the Kolmogorov-Smirnov test.

**Table 3:** Distribution of soil types (ha) and areas of Arabica coffee cultivation in the ten agro-ecological zones of Rwanda.

Agro-Ecological Zone (AEZ)	AEZ label No.	Soil/Coffee coverage	Area per soil type/Area covered by coffee per soil type (ha)^a^								Total	
			ALF	AND	ENT	HIST	INCEPT	MOLL	OX	ULT	ha^b^	%^c^
Buberuka Highlands	6	Soil	2000	99	17 838	10 545	45 835	567	10 492	81 901	167277	7
		Coffee	21	-	157	19	385	7	73	456	1118	3
Central Plateau & Granitic Ridge	7	Soil	68 244	373	47 504	2 219	164 855	1634	48 054	195 007	527 890	23
		Coffee	1306	5	876	30	3286	34	778	3722	10 036	31
Mayaga-Bugesera *(eastern region*)	8	Soil	14 564	-	13 650	28 515	34 038	5768	75 141	37 048	208 722	9
		Coffee	288	-	206	392	599	86	1182	569	3322	10
Eastern Plateau *(eastern region*)	9	Soil	22 782	-	66 166	10 652	76 506	20 420	99 118	76 238	371 881	16
		Coffee	311	-	910	161	1111	294	1459	1021	5268	16
Eastern Savana *(eastern region*)	10	Soil	10 555	-	48 331	38 421	89 951	20 235	186 596	26 586	420 676	18
		Coffee	21	-	118	58	303	26	287	57	870	3

Data were extracted from the Rwanda soil dataset and analyzed using the geo-spatial tools of ArcGIS.

a AND, Andisols; ALF, Alfisols; ENT, Entisols; INCEPT, Inceptisols; HIST, Histosols; MOLL, Mollisols; OX, Oxisols; ULT, Ultisols; VERT, Vertisols.

b Total Rwanda soil and Arabica coffee coverage per agro-ecological zone.

c Soil and Arabica coffee coverage in percentage per agro-ecological zone over total Rwanda soil area and Arabica coffee area, respectively.

**Table 4:** Distribution of soil types (ha) and areas of Arabica coffee cultivation in the ten agro-ecological zones of Rwanda (Cont'd).

Agro-Ecological Zone (AEZ)	AEZ label No.	Soil/Coffee coverage	Area per soil type/Area covered by coffee per soil type (ha)^a^								Total	
			ALF	AND	ENT	HIST	INCEPT	MOLL	OX	ULT	ha^b^	%^c^
Imbo (*Lake Kivu region*)	1	Soil	3349	-	268	-	6489	-	-	3910	14 017	0.6
		Coffee	197	-	16	-	382	-	-	230	825	3
Impara (*Lake Kivu region*)	2	Soil	11 831	-	138	1269	10 111	-	363	40 436	64 147	3
		Coffee	657	-	7	75	558	-	1	2109	3407	11
Kivu Lake Borders (*Lake Kivu region*)	3	Soil	8526	91	12 708	35	25 248	258	253	25 469	72 590	3
		Coffee	328	-	539	2	1085	11	11	1045	3020	9
Birunga/Volcano	4	Soil	1782	47 176	15 450	79	2182	4222	-	3174	74 065	3
		Coffee	16	23	-	-	5	7	-	15	65	0.2
Congo-Nile Watershed Divide	5	Soil	8879	11 613	23 396	5388	124 675	2107	11 341	203 960	391 359	17
		Coffee	112	-	330	8	1553	-	17	2004	4024	13
		Subtotal^d^										
		(ha)	152 513	59 352	245 450	97 123	579 890	55 211	431 358	693 728	2 314 625	
		Subtotal^d^										
		(%)	7	3	11	4	25	2	19	30		100
		Subtotal^e^										
		(ha)	3257	27	3159	743	9266	465	3809	11 228	31 954	
		Subtotal^e^										
		(%)	10	0.1	10	2	29	1	12	35		100

Data were extracted from the Rwanda soil dataset and analyzed using the geo-spatial tools of ArcGIS (cont'd); See Table 4 for the notes.

d Subtotal of soil area per soil type.

e Subtotal of Arabica coffee area per soil type.

**Table 5:** Distribution of slope classes (%) and areas with Arabica coffee in the ten agro-ecological zones of Rwanda.

Agro-Ecological Zone (AEZ)	AEZ label No.	Slope/Coffee coverage	Area per slope category/Area covered by coffee per slope category (ha)			Total	
			<25%	25–55%	>55%	(ha)	(%)^a^
Imbo (*Lake Kivu region*)	1	Slope	10 863	4652	4705	20 220	1
		Coffee	554	238	11	803	3
Impara (*Lake Kivu region*)	2	Slope	45 321	18 165	1407	64 893	3
		Coffee	2417	871	57	3345	10
Kivu Lake Borders (*Lake Kivu region*)	3	Slope	47 780	25 400	413	73 593	3
		Coffee	1911	1016	17	2944	9
Birunga/Volcano	4	Slope	77 170	12 396	1226	90 792	4
		Coffee	59	6	-	65	0.2
Congo-Nile Watershed Divide	5	Slope	229 902	157 739	4258	391 899	16
		Coffee	2059	1922	46	4027	13
Buberuka Highlands	6	Slope	81 303	89 874	5814	176 991	7
		Coffee	435	633	50	1118	3
Central Plateau & Granitic Ridge	7	Slope	369 316	155 345	5070	529 731	22
		Coffee	7212	2944	105	10 261	32
Mayaga-Bugesera *(eastern region*)	8	Slope	214 946	8122	19	223 087	9
		Coffee	3168	152	-	3320	10
Eastern Plateau *(eastern region*)	9	Slope	316 686	63 065	1586	381 337	16
		Coffee	4462	903	24	5389	17
Eastern Savana *(eastern region*)	10	Slope	453 244	25 987	202	479 433	20
		Coffee	641	57	-	698	2
Rwanda - Slope(ha)		Subtotal	1 846 532	560 746	24 701	2 431 979	
(%)			76	23	1		100
Rwanda - Coffee (ha)		Subtotal	22 917	8743	310	31 970	
(%)			72	27	1		100

Data extracted from the digital elevation model (Shuttle Radar Topography Mission at 90×90 m resolution) and analyzed using the geo-spatial tools of ArcGIS.

a Slope and coffee coverage in percentages per agro-ecological zone of the total Rwanda slope and coffee areas, respectively.

The Lake Kivu region (Imbo, Impara, and Kivu Lake Borders zones) in the western province had the highest yields ranging between 0.3 and 3.5 t ha^−1^, with a mean of 1.6 t ha^−1^. This region contained 22% (7127 ha) of the total area cropped with coffee (Table 2). The dominant soil types in the region are Inceptisols and Ultisols, representing ∼6% (2025 ha) and 11% (3384 ha), respectively, of the total area devoted to coffee production (Table 4). Arabica coffee in the region is dominantly cultivated on moderate (<25%) and steep slopes (25–55%) that cover 15% (4882 ha) and 6% (2125 ha), respectively, of the areas under coffee (Table 5). The region is characterized by environmental conditions favorable to coffee production.

Yields in the Birunga (volcano) agro-ecological zone ranged between 0.5 and 2.1 t ha^−1^, with a mean of 1.0 t ha^−1^ (Table 2). The extent of coffee in the zone covered only 65 ha of the land, mainly on Alfisols (16 ha), Andisols (23 ha), and Ultisols (15 ha) (Table 4). Andisols are fertile and productive soils, so the farmers will prefer annual crops over perennial crops such as coffee. Coffee is mainly grown on moderate slopes (Table 5). The effective depth of the soil, dominated by Andisols, is the main factor limiting coffee productivity in the zone.

The Eastern Plateau, Eastern Savanna, Mayaga, and Bugesera zones (i.e. the eastern region) together covered ∼30% (9418 ha) of the total area of coffee production. Yields in the eastern region ranged between 0.3 and 2.2 t ha^−1^, with a mean of 1.0 t ha^−1^ (Table 2). The dominant soil types are Inceptisols, Oxisols, and Ultisols, covering ∼6% (2013 ha), 9% (2928 ha), and 5% (1647 ha), respectively, of the area under coffee (Table 3). Coffee is cultivated on moderate slopes (<25%) that cover more than 26% (8271 ha) of the area with coffee cultivation (Table 5). The dominant infertile soils of the region, very high temperatures, and low rainfall offer limited conditions for coffee productivity.

The Buberuka Highlands and the Congo-Nile Watershed Divide agro-ecological zones are classified as the highlands of the country (above 2000 m a.s.l.). In both zones, coffee was cultivated on ∼16% (5154 ha) of the total coffee area, mainly on moderate (2494 ha, ∼8%) and steep (2555 ha, ∼8%) (Table 5). Yields in the highlands ranged between 0.3 and 3.5 t ha^−1^, with a mean of 1.2 t ha^−1^ (Table 2). Inceptisols and Ultisols are the main soil types, representing ∼6% (1938 ha) and 8% (2460 ha), respectively, of the total area under coffee cultivation (Table 3). Very low temperatures and heavy rainfall limit the productivity of coffee cultivation in the highlands.

### Coffee productivity indices

**Figure 2:**
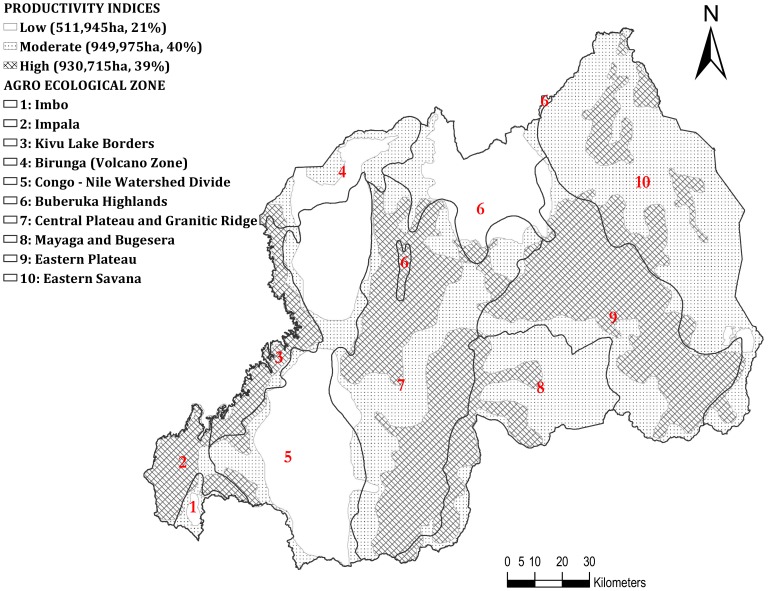
Qualitative Arabica coffee productivity indices (low, moderate, and high) generated by combining factors (elevation, slope, soil type, rainfall, and temperature) using weighted overlay analysis in the ten agro-ecological zones.

Qualitative productivity indices were generated based on soil type, elevation, slope, rainfall, and temperature using weighted overlay analysis (Figure 2). The analysis identified three zones with high, moderate, and low productivity indices representing ∼930 715 (39%), 949 975 (40%), and 511 945 ha (21%), respectively. Approximately 80% of the total area of the country had moderate to high production potential for Arabica coffee. Zones with high potential productivity indices had fertile soils, moderate slopes and altitudes, and favorable climates. The zones with low productivity indices were mainly at high altitudes with high rainfall and low temperatures. The semi-dry eastern regions, where Oxisols and Ultisols are the dominant soil types (Figure 5), have zones with low indices.

**Figure 3:**
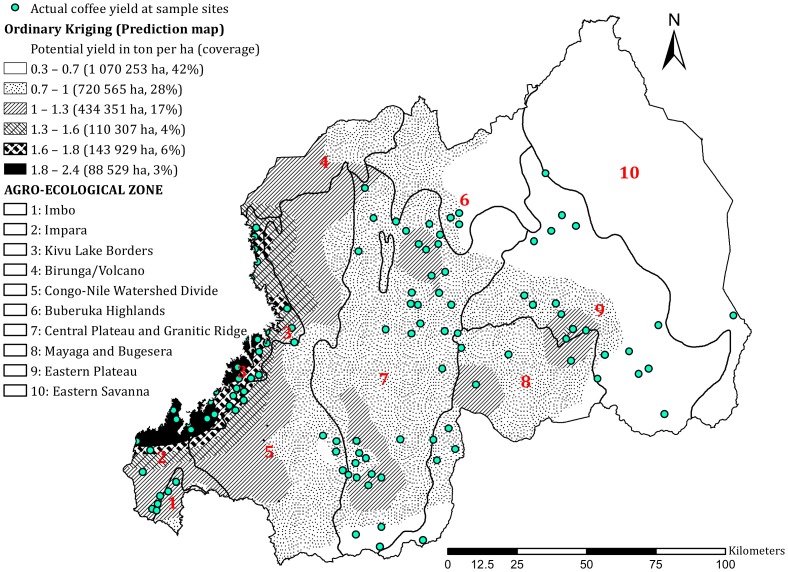
Potential Arabica coffee yield (t ha^−1^) predicted using ordinary kriging in the ten agro-ecological zones based on actual yields (t ha^−1^) measured at sample sites.

**Figure 4:**
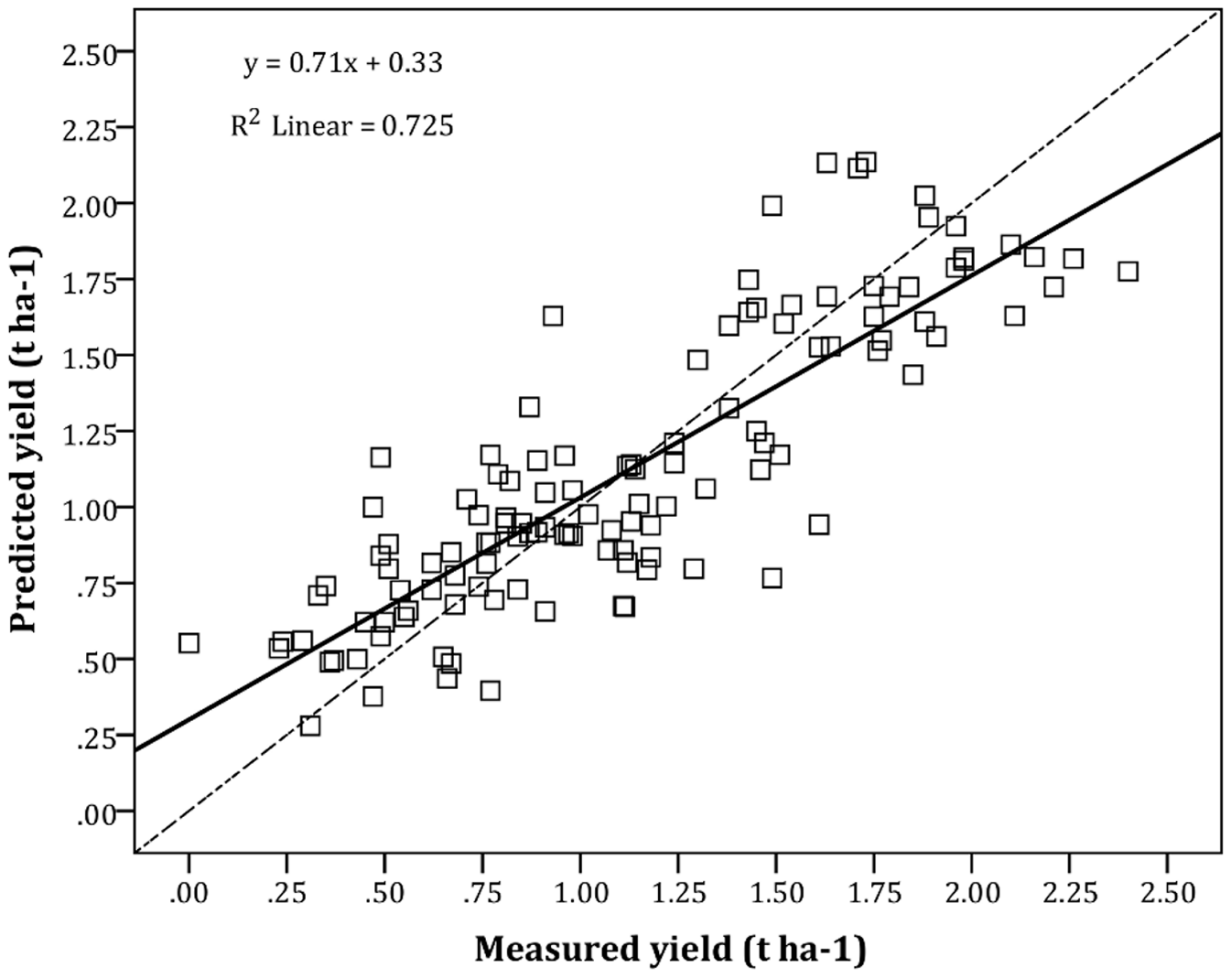
Relationship between measured and predicted Arabica coffee yields – cross validation using ordinary kriging (Predicted Arabica coffee yield index – CYI (t ha^−1^)  =  0.71x + 0.33; Mean Prediction Error – MPE  =  0.0187; Root Mean Square Prediction Error – RMSE  =  0.278; Root Mean Square Standardized Prediction Error – RMSSE  = 0.99; Mean Standardized Prediction Error - MSE  =  0.036; Coefficient of determination – R^2^ = 0.73; Average Standard Error –Avg. SE = 0.291; Sample points, n = 121).

High predicted yields ranged between 1.6 and 2.4 t ha^−1^ along the shores of Lake Kivu and in the Imbo zone (Figure 3). The calculated yields varied between 0.3 and 3.5 t ha^−1^ (Table 2). The prediction map for the country (Figure 3) shows coffee yields varying between 0.3 and 2.4 t ha^−1^. Eighty percent of the country had low yield potentials of 0.3–1 t ha^−1^, whereas 21% of the country had moderate yield potentials of 1.0–1.6 t ha^−1^. The national average yield was predicted to be 1.12 t ha^−1^, and the measured yield (n = 121 sampled sites) was 1.1 t ha^−1^ y^−1^. The correlation between the measured and predicted yields indicated that the prediction model was satisfactory (Coefficient of determination *R* ^2^ = 0.73) (Figure 4).

**Figure 5:**
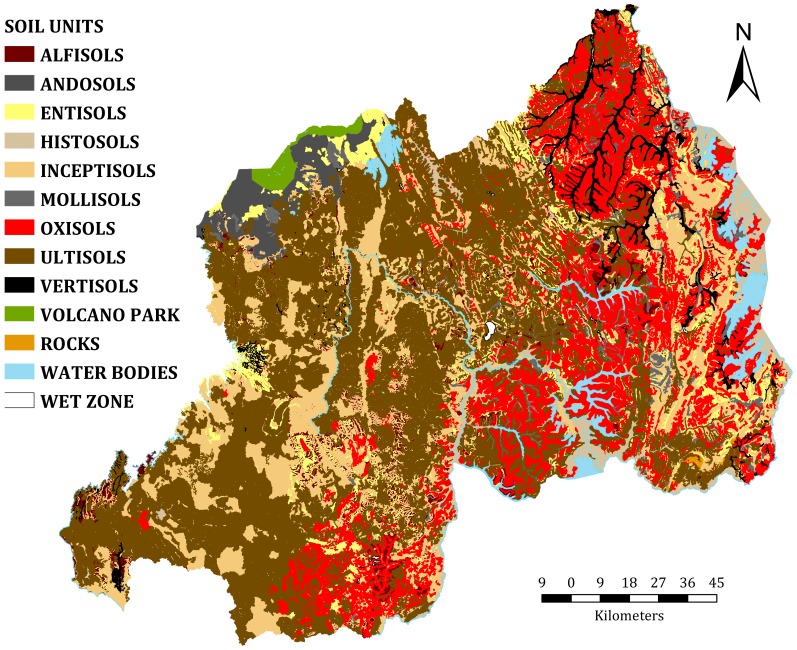
Soil map of Rwanda. Soils are classified using the USDA Soil taxonomy (Source: Data collected from the Ministry of Agriculture and Animal Resources, using the Rwanda soil database) after [32].

## Discussion

Some general information on the suitability of coffee cultivation in Rwanda was available. The spatial variation of coffee production can be explained by the growing conditions, which include biophysical factors such as soil type and properties, parent material, altitude, slope, and climatic conditions. For example, the yield in the Central Plateau and Granitic Ridge zones was limited by soil acidity and the gravel as these soils developed on granitic and schistose materials. On such soils, erosion on slopes above 25% affects the productivity. Similar observations have been made by [33], [34] who indicated that topography can combine with various environmental factors to influence productivity. Topography affects the climate (e.g. variations in temperature and humidity), distribution of soil moisture, soil organic-matter content, soil nutrients, soil textural composition, and soil physical properties, which affect crop growth and yield in a field. The spatial variability of environmental factors can contribute to variability in crop performance, and topography is a vital variable in predicting the spatial variability of crop yields [35].

The multi-criteria overlay analysis used to assess spatial variation in coffee productivity revealed that the Lake Kivu region is an area of high productivity. Similarly, ordinary kriging indicated expected high yields. The Lake Kivu region has favorable soil types and slopes with abundant rainfall and moderate temperatures for the optimal production of coffee. The region has alluvial and very fine clayey soils, developed from basalt, and a high agricultural potential [7].

The Birunga (volcano), Central Plateau, and Buberuka Highlands' zones, the highlands of the Congo-Nile Watershed Divide zone, and the eastern region are areas with both moderate and low productivity indices. The productivity in the Central Plateau zone is mainly limited by infertile soils derived from schistose and granitic materials on the moderately sloped and eroded hillsides. The productivity in the Birunga (volcano) zone is mainly limited by the soil depth (<50 cm) and low temperatures that are sub-optimal for coffee production. High yields were expected in this zone based on the high fertile volcanic soils. Instead, low potential yields were predicted by the kriging model, due to the unsuitable climatic conditions that affect the development and maturity of the berries [36]. In the highlands, mainly in the Buberuka Highlands and Congo-Nile Watershed Divide zones, the production of coffee is limited by very low temperatures, heavy rainfall, and steep slopes that could influence the depletion of soil fertility and reduce yields due to water erosion on the hills. Productivity in the eastern regions is limited by infertile soils and the very high temperatures and low rainfall. Our study demonstrated a decrease in yield in very dry conditions that coincided with lower elevations. Coffee is also constrained by very cold temperatures in the highlands that are often cloudy with low solar irradiation and heavy rainfall. Similar trends of the influence of topography on potato yields have also been reported by [37]. Both the ordinary kriging analysis and the multi-criteria factor analysis thus performed well in assessing and predicting potential yields of coffee. The performance of weighted overlay analysis has also been assessed in cotton by [21]. The relationships of soil, elevation, slope, aspect, and curvature with the stability of crop yields were assessed by [38], who deemed ordinary kriging the best method to estimate crop yield as a function of topography and landscape positions.

A wide range of yields of coffee in Rwanda, varying between 0.8 and 2.8 t ha^−1^ of dry parchment coffee, has also been reported by [6]. The low yields were attributed to coffee variety, agro-ecological conditions, the lack of mineral and organic fertilization, and limited mulching [6]. The national average yield of coffee is estimated at 1.1 t ha^−1^ y^−1^ (this study), but yields above 2.8 t ha^−1^ for dry coffee are rare even with adequate fertilization and sustained crop management [6]. In Uganda, 1.2 t ha^−1^ y^−1^ of dry coffee were recorded for mono-cropped coffee and coffee-banana intercropping systems [39]. The spatial variation in coffee production and productivity are thus mainly influenced by soil properties, soil management, farming practices, and climatic conditions. Similar trends have also been reported by [34], [35].

## Conclusions

The multi-criteria analysis used to assess spatial variation in potential production zones and the productivity of coffee revealed that agro-ecological factors are largely determined suitable zones of coffee productivity. The spatial variation of coffee productivity in the agro-ecological zones was considerable and was influenced by soil properties, soil management, farming practices, and climatic conditions. High production potentials indicated that smallholder farmers could generate income from coffee and could thus improve their livelihoods. In addition, this may provide an opportunity for farmers to purchase more land and extend the area for the production of coffee.

This study demonstrated that both ordinary kriging analysis and multi-criteria weighted overlay analysis performed well for analyzing the spatial distribution and productivity of coffee and for predicting yield. The depletion of soil fertility due to the lack of erosion control in scattered coffee plots on steep slopes is a major factor limiting coffee productivity in Rwanda. The sustainability of coffee productivity could be ensured by intensifying the use of fertilizers, mainly a well-balanced combination of lime, nitrogen, phosphorus, potassium, zinc, and boron. Limited access to financial resources restricts the purchase of these inputs and the use of different types of mulches that can improve soil properties and reduce the erodibility of the soil is recommended.
