# Correction: GIS-based multi-criteria analysis for Arabica coffee expansion in Rwanda

**DOI:** 10.1371/journal.pone.0149239

**Published:** 2016-02-08

**Authors:** Innocent Nzeyimana, Alfred E. Hartemink, Violette Geissen

The DOI associated with the data for the published article is incorrect. The data are available from Figshare at http://dx.doi.org/10.6084/m9.figshare.1128594.
